# The Potential for Spices and Herbs to Improve Dietary Cultural Inclusivity in Healthcare Settings

**DOI:** 10.1093/nutrit/nuaf304

**Published:** 2026-05-26

**Authors:** Minakshi Raj

**Affiliations:** Department of Health and Kinesiology, University of Illinois Urbana–Champaign, Urbana, IL 61801, United States

**Keywords:** McCormick Science Institute, spices and herbs, healthcare, culture, nutrition

## Abstract

Spices and herbs have demonstrated potential for improving health outcomes and the acceptability of healthy dietary options in the general public and in school-aged children. However, the use of spices and herbs in healthcare settings has not been explored or tested. This report outlines preliminary research indicating the need for dietary cultural inclusivity in healthcare settings, and it describes 2 efforts led by researchers at the University of Illinois Urbana–Champaign to improve dietary cultural inclusivity in nutrition services and menu offerings in healthcare settings through increased use of spices and herbs. These efforts have involved expanding awareness and knowledge of spices and herbs, and self-efficacy in using them, among clinicians, patients, and their caregivers through an educational workshop and a novel web-based platform providing nutritional knowledge support.

## BACKGROUND

The US healthcare system is at a critical juncture, as our increasingly multicultural and aging population faces a growing prevalence of diet-related health conditions, such as diabetes and heart disease.^1–6^ About 1 in 4 older adults are members of racial or ethnic communities, and Hispanic Americans, American Indians and Alaska Natives, Black or African-Americans, and certain Asian American subgroups are at a higher risk of diet-related diabetes, heart disease, and complications of these diet-related health conditions.^6–10^ The number of older adults likely to require institutional services, including from hospitals and long-term care settings, is thus expected to grow.^11–13^ Additionally, more than half the population of older adults receive support from people such as friends, neighbors, and relatives, hereon referred to as “caregivers,” who assist with a variety of daily tasks, including grocery shopping and meal preparation.^14^^,^^15^

This article summarizes the findings from a series of our projects, which have indicated the need for dietary cultural inclusivity in healthcare settings. It then describes ongoing approaches to enhancing cultural inclusivity in healthcare food environments by leveraging the use of spices and herbs.

## NEED FOR DIETARY CULTURAL INCLUSIVITY: THE CASE OF OLDER ASIAN AMERICANS

The demands associated with caregiving are well established in the literature, and over 53 million Americans have caregiving responsibilities. Many such caregivers experience a heightened risk of physical and mental distress associated with the time demands and stresses of carrying out caregiving responsibilities.^16–18^ More than half of these caregivers are responsible for preparing meals for an older person; but little is known about the specific responsibilities associated with meal preparation by caregivers, and less is known about the types of resources and interventions that could support caregivers in carrying out these tasks. Using a mixed methods approach involving qualitative interviews (*n* = 40) and a nationwide survey (*n* = 100), we examined the specific case of Asian American family caregivers, seeking to understand their experiences and the challenges with managing the older person’s diet-related needs and in navigating their particular social and cultural norms. Diet-related responsibilities were the most common challenge reported, and participants described 2 significant issues associated with diet-related support.^19^ First, caregivers were often responsible for implementing dietary recommendations provided by clinicians (eg, physicians or dietitians) in the home for a relative; yet, these recommendations were typically rooted in Western/American dietary norms and patterns, while their older relatives preferred traditional meals. Some caregivers had taken additional time to learn about concepts of nutrition and were able to successfully reduce or completely eliminate the need for long-term medications such as those used for diabetes while maintaining their relative’s traditional dietary preferences. Second, in this particular sample, enrollment in long-term care facilities was low due to cultural norms and stigma around institutional healthcare. Among those caregivers with relatives receiving institutional care in hospitals or long-term care settings, many were responsible for preparing and delivering traditional meals to their older relative(s) in facilities that only offered Western/American dietary options. Those who were considering enrolling their relative in long-term care settings were concerned about the food available in those facilities and expressed concerns about having observed refusal to eat and unintentional weight loss. Our findings made salient the urgent need to enhance the healthcare system—both in terms of the delivery of nutrition services and food service offerings—to meet the needs of culturally diverse older adults and their family caregivers.

## INSTITUTIONAL FACILITATORS AND BARRIERS TO DIETARY CULTURAL INCLUSIVITY

**Figure 1.**
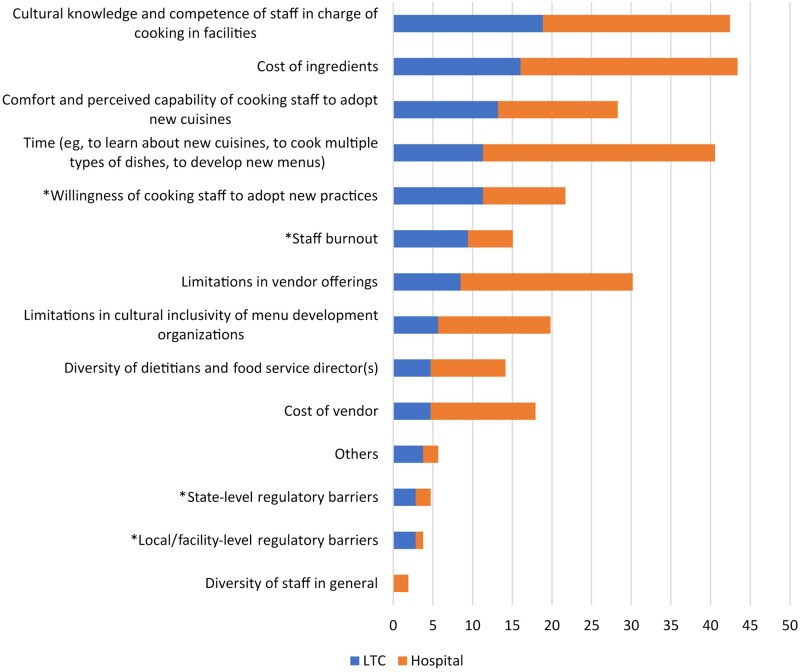
Perceived Barriers to Implementing Culturally Inclusive Foods in long-term care (Raj et al 2023). *denotes barriers mentioned more frequently by long-term care than hospital respondents Figure with permission from Raj et al (2023) Perceived Facilitators and Barriers to Implementing Culturally Inclusive Diets into Hospitals and Long-Term Care Facilities. Journal of the American Medical Directors Association. doi: 10.1016/j.jamda.2023.04.018

To better understand the potential for transforming our approach to food within the US healthcare system, we conducted a survey with Registered Dietitian Nutritionists (RDNs) and food service directors working in healthcare facilities across the United States in 2022 to understand their perceptions of facilitators and barriers to implementing culturally diverse cuisines in healthcare settings.^20^ The most common barrier reported across the full sample (*n* = 118) was lack of cultural knowledge and awareness of staff in preparing culturally relevant meals in these facilities (Figure 1). This is consistent with studies that find that dietetics programs are limited to an immersive course in just a few culinary traditions and do not necessarily enable enough time for learning about a variety of cultural and culinary traditions.^21^ Our preliminary studies, in addition, pointed to the need for educational interventions within healthcare organizations to supplement the existing community-based programs and resources for older adults and their caregivers. In other words, resources to improve education and access to information about culturally diverse cuisines at the point-of-care are currently lacking, and this has informed our current work with the support of the McCormick Science Institute.

## APPROACHES TO IMPROVING DIETARY CULTURAL INCLUSIVITY IN HEALTHCARE

Our approach to improving dietary cultural inclusivity involves long-term effort that prioritizes education and awareness. Policy change is ultimately required for sustainable and scalable changes in our approach to food in healthcare settings^22^; however, influencing policy requires robust evidence concerning the effectiveness of various approaches in terms of positive impacts on costs, quality of care, and patient outcomes. Thus, our current work involved the development and testing of 2 interconnected interventions that sought (1) to improve the awareness and knowledge of spices and herbs, and self-efficacy in using them among RDNs and caregivers; (2) to enhance the rapport and shared decision-making between patients, caregivers, and RDNs; and (3) to build organizational capacity to deliver culturally relevant food and nutrition services and training for patients and their caregivers. The 2 interventions included (1) an educational workshop on spices and herbs delivered to dietitians and food service personnel in long-term care facilities and hospitals across Illinois, and (2) the development of a novel web-based platform to *Support Personalized and Inclusive Cuisines in Environments for Healthcare (SPICE-Healthcare)*, which features an interactive *spices and herbs explorer*.

### Educational Workshop on Spices and Herbs

A considerable body of literature illustrates the extensive health benefits of many culinary spices and herbs found in culturally diverse dietary traditions, including anti-hypertensive, anti-inflammatory, and anti-hyperlipidemic properties of spices and herbs.^23–26^ Studies also demonstrate efficacy in leveraging spices and herbs to increase vegetable intake, and that the use of spices and herbs can enhance consumer acceptability and liking of low-sodium or reduced-fat foods.^27–36^ Additionally, the creative repurposing of food can reduce the significant amount of food waste generated in healthcare facilities, and educating staff in how to incorporate underused spices and herbs in the preparation of existing food options could be an effective and cost-saving strategy for healthcare facilities.^37^^,^^38^

In the fall of 2023, we developed and administered a survey with over 200 RDNs, physicians, chefs, and food service workers working in healthcare settings to learn about their awareness and knowledge of culturally diverse spices and herbs, and self-efficacy in using them (*n* = 218). We sought to learn about the participants’ use of spices and herbs in their personal cooking; for chefs and food service workers, we also gathered information on their professional food preparation approaches. A variety of culinary traditions were reflected in the participants’ personal cooking, and they were generally aware of most of the 46 spices and herbs included in our survey. For instance, more than 50% of respondents typically cooked Latin American (58.3%), Asian or Middle Eastern (70.6%), African (70.6%), and/or North American cuisine (80.7%). However, actual use of a large number of spices and herbs was low relative to awareness, suggesting that participants may not be familiar with how to prepare food with these spices and herbs. Moreover, nearly all respondents reported using spices and herbs to make their food taste better, but only about half (50.5%) reported incorporating spices and herbs for their added health benefits.

In the spring of 2025, we began developing an educational workshop on spices and herbs that will be hosted by a continuum of care retirement community (CCRC) in Urbana, IL, in 2025. The educational workshop will be facilitated by the author (M.R.) and a trained chef who will provide a historical overview of spices and herbs, including the origins of the various spices and herbs. This will also involve a cooking demonstration and tasting led by the chef featuring versatile, easily accessible ingredients often used in long-term care facilities but prepared with spices and herbs typical of Latin American and Asian American culinary traditions. We expect at least 40 RDNs, chefs, and food service staff from across Illinois to attend the session and to complete pre- and post-workshop surveys, enabling us to understand the efficacy of such a workshop in raising the knowledge and awareness of herbs and spices, and in building self-efficacy in the use of herbs and spices to bring new culinary traditions and flavors into healthcare settings.

As a pilot test, we conducted a small workshop at this CCRC in the summer of 2024 with a group of 10 residents who tested 4 versions of rice, potatoes, tomatoes, cucumbers, and plain yogurt seasoned with spices and herbs from around the world, prepared by the facility’s chef (Table 1). Residents also played “spice jeopardy,” in which they tested their knowledge of spices and herbs from around the world. This event was a success; one resident remarked about the potatoes seasoned with berbere: *“I don’t usually like potatoes, but if they were made this way, I’d eat them all the time!”* Other residents sought second and third servings of our plain yogurt dish that was flavored with allspice.

**Table 1.** Spices and Herbs Used in a Workshop with Residents at a Local CCRC in Urbana, IL

Cuisine	Spices and herbs used	Paired with …
Mediterranean	Oregano Parsley Dill	Tomato and cucumber salad
Asian	Brown mustard seeds Ginger	
European	Paprika Black pepper	
Indian^a^	Cumin Turmeric Black pepper	Rice
Japanese	Furikake	
Mediterranean	Lemon zest Dill Black pepper	
Mediterranean	Rosemary Thyme	Potatoes
African	Berbere	
Middle Eastern^a^	Cumin Black pepper	
Asian	Allspice	Plain yogurt

a Only these recipes included <1/4 teaspoon of salt; other recipes did not include any salt.

*Abbreviation:* CCRC, continuum of care retirement community

### Supporting Personalized and Inclusive Cuisines in Environments for Healthcare

Our second effort involves the development of a novel web-based platform to support culturally relevant nutrition services in healthcare settings.^39^ This work is responsive to the findings from our preliminary work that highlighted how Western/American dietary recommendations were misaligned with the traditional preferences of older adults in the home; and the distress associated with preparing meals, or reluctance to enroll in institutional healthcare, due to limitations in menu offerings.

Supporting Personalized and Inclusive Cuisines in Environments for Healthcare (SPICE-Healthcare) follows the Nutrition Care Process^40^ and provides a culturally and medically tailored comprehensive nutrition assessment and educational resource hub with recipe videos, accessible resources about various diet-related health conditions, and a spices and herbs interactive explorer for clinicians, patients, and caregivers of all ages to learn about the history, origins, and uses of spices and herbs from around the world. We have fully programmed and tested the comprehensive nutrition assessment tool with 30 RDNs, who reported above-average usability and acceptability of the tool, and who gave positive qualitative feedback about the opportunity to learn about multiple cultures and to adapt the tool to meet the needs of diverse patients and their caregivers. Next year, we plan to program and test the interactive *spices and herbs explorer* tool with clinicians, patients, and their caregivers, to better understand its utility and usability in clinical and community settings.

## CONCLUSION

Daniel Gilbert says “the secret of happiness is variety, but the secret of variety, like the secret of all spices, is knowing when to use it.” Our work with the McCormick Science Institute builds on evidence gathered over generations about the benefits, utility, and characteristics of spices and herbs and seeks to translate these benefits to healthcare settings and nutrition service delivery.^41–45^ Our studies find that there is a need for raising awareness, knowledge, and self-efficacy related to preparing meals and providing guidance to diverse populations with traditional food preferences in healthcare settings. Our studies also illustrate that, with comprehensive and useful tools and resources, there is potential for incorporating increased and informed use of spices and herbs into healthcare settings. This can support a sense of belonging, and the opportunity for using creativity and imagination in the clinicians, food service staff, chefs, patients, and caregivers in our healthcare system. Future studies can evaluate the effectiveness of these interventions in terms of quality of care; mental, physical, and social health outcomes; and costs associated with care.

## Data Availability

The data underlying this article cannot be shared publicly for the privacy of individuals that participated in the study. The data will be shared on reasonable request to the corresponding author.
